# Cognitive Impairments and Self-Reported Sleep in Early-Stage Parkinson’s Disease with Versus without Probable REM Sleep Behavior Disorder

**DOI:** 10.3390/brainsci10010009

**Published:** 2019-12-21

**Authors:** Jonathan Trout, Taylor Christiansen, M. Brooks Bulkley, Jared J. Tanner, Christopher N. Sozda, Dawn Bowers, Daniel B. Kay

**Affiliations:** 1 Department of Psychology, Brigham Young University, Provo, UT 84602, USA; jonathan.trout2015@gmail.com (J.T.); taylor7832@gmail.com (T.C.); brooksbulkley@gmail.com (M.B.B.); 2 Department of Clinical and Health Psychology, University of Florida, Gainesville, FL 32611, USA; jjtanner@gmail.com (J.J.T.); dawnbowers@phhp.ufl.edu (D.B.)

**Keywords:** REM sleep behavior disorder, Parkinson’s disease, sleep quality, executive control, cognition, verbal fluency

## Abstract

Parkinson’s disease (PD) is associated with cognitive and sleep impairments. The presence of rapid eye movement (REM) sleep behavior disorder (RBD) symptoms may represent a worse disease prognosis for PD individuals. We investigated cognitive functioning and self-reported sleep in early-stage PD individuals with (*n* = 19) or without (*n* = 31) probable RBD. Probable RBD was defined as >5 on the REM Sleep Behavior Disorder Screening Questionnaire. Inhibition, visuospatial cognitive abilities, working memory, sustained visual attention, verbal fluency, and episodic memory were assessed. Sleep impairments were assessed using the Pittsburgh Sleep Quality Index, Insomnia Severity Index, Epworth Sleepiness Scale, and Patient-Reported Outcomes Measurement Information System questionnaires. Chi-squared, Mann-Whitney *U*, and independent sample *t*-tests were employed to assess group differences. Participants with PD and probable RBD performed significantly worse on word reading and switching verbal fluency tasks than PD participants without probable RBD (*p* < 0.05). No significant differences were found in mood, PD severity, or sleep measures between PD individuals with or without probable RBD. Cognitive tasks that involve verbal or switching components may be most impaired in PD individuals with probable RBD. Larger samples are needed to determine whether other cognitive domains and sleep features are significantly associated with RBD in PD.

## 1. Introduction

Parkinson’s disease (PD) is a common neurodegenerative movement disorder characterized by resting tremor, rigidity, bradykinesia, and postural instability. As a complex, multifaceted disorder, PD is associated with cognitive impairment [1,2,3,4,5,6,7] and sleep problems [8,9,10,11,12]. Rapid eye movement (REM) sleep behavior disorder (RBD) affects up to 37–47% of PD patients [7,13] and is one of the most common and distressing sleep disorders associated with PD. The loss of muscle atonia during REM sleep in RBD and “acting out” dreams can lead to injury for PD individuals and their bed partners [14]. The presence of RBD in PD may represent a more severe disease prognosis associated with accelerated cognitive decline and poorer sleep [15,16,17].

Several studies have investigated the impact of RBD on cognitive functioning in PD [18,19,20,21,22]. While differing in the domains examined and the measures used, these studies identified that RBD in PD is most consistently associated with poorer performance on executive functioning, attention, verbal learning and memory, and visuospatial processing tasks [18,19,20,23]. One study found that RBD in PD was not associated with poorer performance on nonverbal learning and memory tasks [18], suggesting a potential link to unique verbal deficiencies in PD patients with RBD. Understanding the connection between specific cognitive impairments associated with RBD in PD patients may elucidate their pathophysiology and lay the groundwork for effective treatments of these impairments.

The impact of RBD on sleep quality has been a common question in the literature. Some studies have investigated the impact of RBD on polysomnographic sleep measures and found that RBD was associated with more night-time awakenings and decreased sleep duration, particularly in REM sleep [14,21,24]. However, other studies found that RBD was not associated with some sleep measures, such as sleep latency and sleep efficiency [14,25]. The effect of RBD on self-reported sleep measures in PD patients is limited primarily to measures of daytime sleepiness, insomnia severity, and a PD sleep scale [18,19,20,24,26]. Most studies found no association between daytime sleepiness or insomnia severity with RBD in PD patients [18,19,20,26], but some found an association with daytime sleepiness or the PD sleep scale [24,26]. When a study investigated the relationship between idiopathic RBD (iRBD) and subjective sleep measures, iRBD participants reported greater insomnia severity and sleep disturbance, suggesting RBD as a potential contributor to sleep problems [27]. Investigating subjective sleep across a wider variety of sleep measures in PD patients with RBD may elucidate specific self-reported sleep problems associated with RBD and could shed light on the mixed findings in the literature.

The purpose of this analysis was to investigate whether individuals with probable RBD and early-stage PD (*n* = 19) exhibit greater cognitive impairment than PD individuals without RBD symptoms (*n* = 31). We hypothesized that the combination of early-stage PD and probable RBD would result in greater cognitive impairment. We also explored whether individuals with PD who had probable RBD had poorer self-reported sleep across a wide variety of sleep features compared to PD individuals without probable RBD.

## 2. Materials and Methods

### 2.1. Participants

This study is a secondary analysis of data collected to investigate the impact of PD on sleep, cognition, and mood. Participants with PD were categorized as having probable RBD based on a REM Sleep Behavior Disorder Screening Questionnaire (RBDSQ) score greater than 5 [28]. Demographic and sleep features of this sample have been reported previously [29]. Most participants were older adults (*Mdn* = 66.5), white (76%), and male (76%). The groups were similar in demographic characteristics, except for age; probable RBD group participants were significantly older than those without probable RBD.

Participants were recruited from the University of Florida (UF) Center for Movement Disorders and Neurorestoration (CMDN). The Unified Parkinson Disease Rating Scale (UPDRS) [30] was used to clinically diagnose idiopathic PD. Participants presented with early-to-middle stage PD based on a Hoehn–Yahr staging score (*M* = 1.8, *SD* = 1.1) [31]. The disease information for PD participants was obtained from a clinical research database maintained by the UF CMDN. This study was conducted with the evaluation and approval of the UF Institutional Review Board (IRB-01# 117-2011). Exclusion criteria for the study were: (1) the inability to give informed consent, (2) lower than an 8th grade education, (3) evidence of significant neurological problems other than PD (tumors, epilepsy, etc.), (4) significant medical problems other than PD such as myocardial infarction (<6 months), congestive heart failure (functional stage >3), etc., (5) a severe psychiatric disease including a prior history of substance abuse disorder, psychosis, mania, obsessive compulsive disorder, attention deficit disorder, and post-traumatic stress disorder or a current severe major depressive disorder determined by a score >19 on the Patient Health Questionnaire-9 [32], (6) a reduced mental status based on scores <25 on the Mini-Mental State Exam [33], (7) an inability to read and comprehend English, (8) a history of neurosurgery, (9) diagnosis, treatment, or bed partner reports suggestive of obstructive sleep apnea, restless leg syndrome, or periodic limb movement disorder, and (10) a lack of a bed partner able to document the absence of these sleep disorder symptoms.

### 2.2. Measures

#### 2.2.1. Overview

Participants completed a screening survey, a semi-structured clinical sleep interview, several sleep- and mood-related questionnaires, and a selected battery of neuropsychological measures in private testing rooms at UF or in the participants’ home. Anxiety and mood were assessed using the State-Trait Anxiety Inventory [34] and the Patient Health Questionnaire-9 [32], respectively. Severity of PD was measured using the Unified Parkinson’s Disease Rating Scale and PD duration, which was measured in months. Neuropsychological testing occurred while PD individuals were medicated. This study compared groups (PD with vs. without probable RBD) across several demographic, mood, PD severity, cognitive, and sleep variables.

#### 2.2.2. Cognition Measures

The Color-Word Interference Test of the Delis-Kaplan Executive Functioning System (D-KEFS) was employed to evaluate cognitive response inhibition [35]. The scores were converted to scaled-score equivalents according to age norms using the D-KEFS manual. Lower scores on the inhibition task indicated lower cognitive inhibition. The Judgment of Line Orientation (JOLO) test was used to evaluate visuospatial processing [36]. Scores were converted to scaled scores according to age norms [37]. Higher scores indicated better visuospatial performance. Working memory was assessed using the average accuracy and response time on the 2- and 3-back conditions of a computerized n-back task [38]. Higher accuracy scores and lower response times indicated better working memory. A continuous performance task (CPT) was used to evaluate sustained visual attention [39]. Lower response times and higher accuracy scores indicated better sustained attention. Cognitive-shifting was evaluated using the category switching condition of the D-KEFS Verbal Fluency test. Higher scores on the switching task were conceptualized as better cognitive-shifting. A story memory test, modeled after the Logical Memory subtest of the Wechsler Memory Scale (WMS III, IV), was employed to assess episodic memory [40]. Immediate and 30-minute-delayed responses were recorded after two stories were read to the participants. Due to an experimenter error, one participant’s performance was not recorded. Higher scores on this test indicated better episodic memory.

#### 2.2.3. Sleep Measures

The total score on the Pittsburgh Sleep Quality Index (PSQI) was used to assess subjective sleep quality [41]. Item 4 of the PSQI was used to assess average subjective total sleep time normally obtained each night over the previous month [42]. Higher total sleep time and global PSQI indicated better sleep quality. The Insomnia Severity Index (ISI) was used to assess the sleep quality and insomnia severity over the previous 2 weeks [43]. Higher ISI scores indicated higher insomnia severity and lower sleep quality. The Epworth Sleepiness Scale (ESS) was employed to provide an index of the severity of daytime sleepiness [44]. Higher ESS scores indicated higher daytime sleepiness. The Patient-Reported Outcomes Measurement Information System Sleep Disturbance (PROMIS-SD) and the Sleep-Related Impairment (PROMIS-SRI) questionnaires were used to assess sleep disturbance and sleep-related impairment, respectively [45]. Higher scores on these questionnaires represented greater sleep disturbance and sleep-related impairment, respectively.

### 2.3. Analyses

All variables were assessed for normality within each group using the Shapiro-Wilk test and visual inspection of histogram distributions. Statistical significance was set at *p* < 0.05. Chi-squared test, Mann-Whitney *U* test, or independent sample *t*-tests were used to compare demographic, mood, PD severity, cognition, and sleep measures in PD participants with probable to PD participants without probable RBD. IBM SPSS 26 statistical software was used to analyze these data. Effect sizes were calculated using *ϕ* for measures compared using 2 × 2 contingency table Chi-squared tests, Cramer’s *V* for measures compared using non-2 × 2 contingency Chi-squared tests, Spearman’s Rho (*r_s_*) for measures compared using Mann-Whitney *U* tests, and Cohen’s *d* for measures compared using independent sample *t*-tests. Pirate plots created in R were used to visualize group differences in variables with moderate-to-strong effect sizes (*ϕ* > 0.3, Cramer’s *V* > 0.13 for *df* = 5, *r* > 0.3, and Cohen’s *d* > 0.5).

## 3. Results

### 3.1. Probable RBD and Cognition Analyses

**Figure 1:**
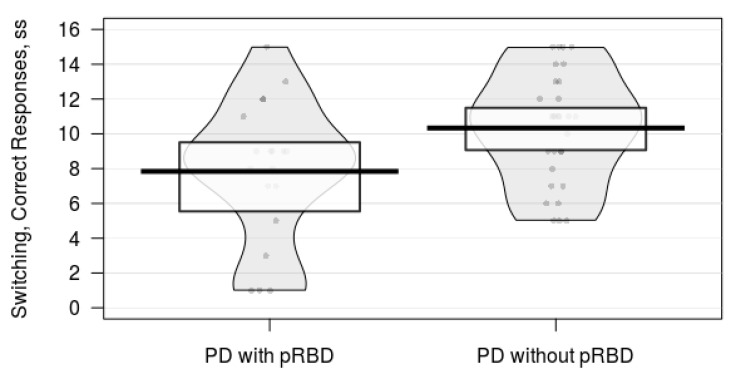
A pirate plot comparing category switching verbal fluency task performance between Parkinson’s disease (PD) individuals with and without probable rapid eye movement sleep behavior disorder (pRBD). The bars represent the means of each group. The points represent raw data. The bands around each bar represent 95% confidence intervals. The beans represent the smoothed density curve of the data distribution. The PD with pRBD group tended to perform worse on the category switching task than the PD without pRBD group did.

**Figure 2:**
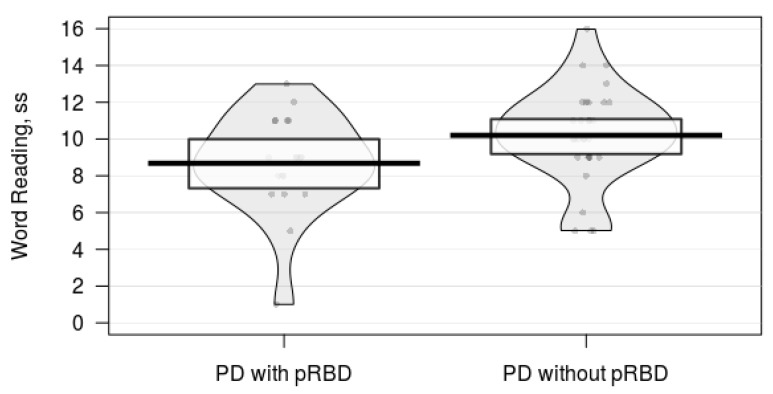
A pirate plot comparing word reading task performance between Parkinson’s disease (PD) individuals with and without probable rapid eye movement sleep behavior disorder (pRBD). The bars represent the means of each group. The points represent raw data. The bands around each bar represent 95% confidence intervals. The beans represent the smoothed density curve of the data distribution. The PD with pRBD group tended to have a worse performance on the word reading task than the PD without pRBD group did.

Participants with probable RBD had poorer performance on verbal fluency category switching and word reading tasks than participants without probable RBD (Table 1). There were no significant group differences between the PD with probable RBD and the PD without probable RBD groups on tasks of cognitive inhibition, visuospatial processing, working memory, sustained attention, or episodic memory (Table 1). The pirate plots and effect sizes suggest that PD participants with probable RBD tended to have a worse performance on category switching verbal fluency (Figure 1) and word reading tasks (Figure 2).

### 3.2. Probable RBD and Demographic, Sleep, Mood, and PD Severity Analyses

Age was the only demographic variable that significantly differed between the two groups (*p* > 0.05; Table 1). No significant group differences were found in the sleep, mood, or PD severity measures between the PD without probable RBD and the PD with probable RBD groups (Table 1).

## 4. Discussion

In this study, we investigated whether individuals with PD co-morbid with probable RBD had greater cognitive and sleep problems than those with PD who did not have probable RBD. We found that PD individuals with probable RBD had significant deficits in word reading and switching verbal fluency tasks compared to PD individuals without probable RBD. Scores on these tasks were scaled to age norms to eliminate the potential confound of age differences across groups. This cognitive finding agrees with the conclusions of previous studies that suggest an association between RBD and verbal task performance in PD individuals [18,19,20]. It has already been reported in some studies that PD patients demonstrate significant cognitive deficits in cognitive shifting and verbal fluency tasks [46,47], suggesting that RBD is associated with additional verbal task impairment in PD. Differing from previous studies, however, we found no significant differences between PD with or without probable RBD participants on the performance of attention, memory, and visuospatial tasks [18,19,20]. This discrepancy is not likely due to the advanced age of the PD and probable RBD group or the similar level of PD severity of the two groups, since other studies have included comparable sample descriptions while reporting that other cognitive domains were affected [16,25,48].

We failed to detect significant differences between PD participants with or without probable RBD on self-reported sleep measures. These findings align with previous studies that suggest that PD patients with RBD do not report worse sleep problems than PD patients without RBD do [18,19,20,23,24,25]. These findings, however, conflict with previous studies that found increased daytime sleepiness and lower sleep quality in PD patients with RBD compared to those without RBD [49,50]. We note that there was a trend association suggesting that probable RBD participants may have greater sleepiness than participants without probable RBD. Again, the inability of our study to detect significant sleep differences in our group may be due to our relatively small sample size.

These results may be explained mechanistically by RBD in PD being associated with greater impairment in frontal and temporal brain circuitry, specifically with connections implicated in verbal executive functions that are unrelated to PD-specific pathology [51,52]. As neurodegeneration progresses, RBD may amplify verbal impairments found in PD, particularly those that involve switching, that already exist. In particular, since related studies have also reported verbal impairments, such as in fluency, learning, and memory tasks [18,19,20], these data may represent one of the most unique cognitive impairment symptoms that result from RBD in PD. Other cognitive impairments may then develop as neurodegeneration progresses in parallel or serially to regions responsible for other cognitive tasks [15].

Our findings must be considered in the context of several study limitations. The relatively small sample size of this study, particularly for the probable RBD group, and the large number of variables, limited the power of our findings. Our results may also have been confounded by the presence of comorbidities of PD that were not addressed in this study and that may have impacted the cognitive performance and sleep quality of study participants. By screening for dementia, our study may underestimate the detrimental effects of PD and RBD on cognition due to selection effects [19]. Prospective studies may provide valuable insights not possible with a cross-sectional design. Additionally, study participants were relatively young for their disease duration, which suggests that they may have a less severe PD presentation than may be found in similar studies. Because of this, these results cannot be generalized to PD individuals with a greater disease severity.

## 5. Conclusions

In recent years, studies have identified RBD as an important prodrome and comorbidity of PD. This study adds to the literature focused on understanding the detrimental effects of RBD on cognition and self-reported sleep impairment in early-stage PD patients. The results of this study suggest that RBD in early-stage PD is most strongly associated with verbal tasks involving word reading or switching verbal fluency, difficulties that exceed those seen in PD patients without RBD. These cognitive deficits may represent some of the more unique impairments associated with RBD in PD and could potentially be targeted by therapies to ameliorate these deficits.

## Figures and Tables

**Table 1:** Demographic, mood, Parkinson’s disease (PD) severity, cognition, and sleep measures in PD individuals with or without probable rapid eye movement sleep behavior disorder (pRBD).

	PD with pRBD (*n* = 19)	PD without pRBD (*n* = 31)	*χ* ^2^ */Z/t*	*df*	*p*-Value	Effect Size (*ϕ/V/r_s_/d*)
**Demographic, Mood, and PD Measures**						
Age, y	70.1 (7.4)	65.7 (6.6)	−2.16	48	0.036 *	*d* = −0.62
Male sex, *n*	16	22	1.13	1	0.760	*ϕ* = 0.15
Education, y	16 [13, 20]	16 [12, 18]	−0.84		0.400	*r_s_* = −0.12
White, *n*	16	22	0.13	1	0.721	*ϕ* = 0.05
Married, *n*	15	28	5.71	5	0.335	*V* = 0.15
State-Trait Anxiety Inventory, State	25 [20, 41]	35 [27, 43]	1.69	-	0.091	*r_s_* = 0.24
Patient Health Questionnaire-9	5 [3, 8]	6 [2, 10]	0.20	-	0.841	*r_s_* = 0.03
Modified Hoehn-Yahr Scale ^†^	2 [2, 2]	2 [2, 2]	0.92	-	0.358	*r_s_* = 0.13
PD duration, mos ^‡^	84 [36, 120]	96 [66, 135]	0.73	-	0.330	*r_s_* = 0.12
**Cognition Measures**						
D-KEFS Color-Word Interference Test						
Color naming, ss	8.7 (2.5)	9.7 (2.4)	1.42	48	0.162	*d* = 0.41
Word reading, ss	8.7 (2.7)	10.3 (2.6)	2.06	48	0.044 *	*d* = 0.60 ^§^
Inhibition, ss	9.2 (3.1)	10.0 (3.7)	0.80	48	0.425	*d* = 0.23
Switching, ss ^‡^	9.4 (3.5)	10.8 (2.4)	1.65	46	0.106	*d* = 0.48
Visuospatial processing, JOLO, ss	12.2 (2.8)	12.2 (2.4)	0.01	48	0.996	*d* = 0.00
Working Memory, 2- & 3-back						
Average accuracy ^†‡^	0.8 [0.8, 0.9]	0.9 [0.8, 0.9]	−0.71		0.480	*r_s_* = 0.10
Average RT, ms ^†‡^	876 [781, 875]	902 [768, 902]	−0.48		0.633	*r_s_* = 0.07
Sustained Attention, CPT						
Total accuracy ^†^	0.9 [0.6, 1.0]	0.9 [0.8, 1.0]	−0.51	-	0.608	*r_s_* = 0.04
Total RT, ms ^†^	399 [374, 476]	394.0 [357, 455]	−0.26	-	0.797	*r_s_* = −0.07
D-KEFS Verbal Fluency Test						
Letter, ss ^†^	10.4 (3.4)	11.2 (3.7)	0.70	47	0.485	*d* = 0.20
Category, ss ^†^	9.6 (2.3)	11.0 (3.3)	1.66	47	0.104	*d* = 0.48
Switching, ss ^†^	7.8 (4.1)	10.3 (3.2)	2.38	47	0.021 *	*d* = 0.69 ^§^
Episodic memory, stories						
Immediate recall ^†^	20.0 (6.1)	21.0 (4.8)	0.59	47	0.561	*d* = 0.17
Delayed recall ^†^	17.0 (5.4)	17.0 (4.5)	0.19	47	0.854	*d* = 0.06
**Sleep Measures**						
Pittsburgh Sleep Quality Index total score	6.9 (5.2)	7.1 (3.2)	0.15	48	0.878	*d* = 0.04
Total sleep time, min	396 (105)	401 (65)	0.23	48	0.816	*d* = 0.24
Insomnia Severity Index total score	8 [3, 15]	6 [3, 12]	−0.84	-	0.399	*r_s_* = −0.12
Epworth Sleepiness Scale total score	10 [7, 16]	8 [4, 9]	−1.80	-	0.073	*r_s_* = −0.26
Sleep disturbance, PROMIS-SD ^†^	53 [41, 77]	52 [45, 59]	−0.51	-	0.608	*r_s_* = −0.07
Sleep-related impairment, PROMIS-SRI ^†^	31 [27, 49]	34 [26, 38]	−0.81	-	0.417	*r_s_* = 0.12

Note. Data that were not normally distributed are reported as *Mdn* [*IQR*]. Percentiles were determined using weighted averages. Normally distributed data are reported as *M*(*SD*). ^†^ indicates data missing for one participant. ^‡^ indicates data missing for two participants. ^†‡^ indicates data missing for three participants. * indicates that the PD with pRBD and PD without pRBD groups differed significantly from each other at the *p* < 0.05 level. *Φ*: Mean Square Contingency Coefficient. *V*: Cramer’s *V*. *r_s_*: Spearman’s Rho. *d*: Cohen’s *d*. ^§^ indicates a medium effect size. ss: scaled score equivalent based on age norms. D-KEFS: Delis-Kaplan Executive Functioning System. JOLO: Judgment of Line Orientation. RT: response time. CPT: Continuous Performance Task. PROMIS-SD: Patient-Reported Outcomes Measurement Information System-Sleep Disturbance. SRI: Sleep-Related Impairment.
